# Exploring the Oxidation of Lignin-Derived Phenols by a Library of Laccase Mutants

**DOI:** 10.3390/molecules200915929

**Published:** 2015-09-02

**Authors:** Isabel Pardo, Susana Camarero

**Affiliations:** Centro de Investigaciones Biológicas (CSIC), Ramiro de Maeztu 9, 28040 Madrid, Spain; E-Mail: iparmen@cib.csic.es

**Keywords:** lignin-derived phenols, laccase, substrate binding pocket, saturation mutagenesis, multi-screening assay

## Abstract

Saturation mutagenesis was performed over six residues delimiting the substrate binding pocket of a fungal laccase previously engineered in the lab. Mutant libraries were screened using sinapic acid as a model substrate, and those mutants presenting increased activity were selected for exploring the oxidation of lignin-derived phenols. The latter comprised a battery of phenolic compounds of interest due to their use as redox mediators or precursors of added-value products and their biological activity. The new laccase variants were investigated in a multi-screening assay and the structural determinants, at both the substrate and the protein level, for the oxidation of the different phenols are discussed. Laccase activity greatly varied only by changing one or two residues of the enzyme pocket. Our results suggest that once the redox potential threshold is surpassed, the contribution of the residues of the enzymatic pocket for substrate recognition and binding strongly influence the overall rate of the catalytic reaction.

## 1. Introduction

Laccases are blue-multicopper oxidases that are widely distributed in nature. They primarily catalyze the oxidation of substituted phenols and aromatic amines, among other aromatic compounds, coupled to the reduction of O_2_ to water. Laccases participate in processes of polymerization, such as lignin formation in plants, cuticle sclerotization in insects, and sporulation and pigmentation in bacteria and fungi; and depolymerization, mainly lignin oxidation by fungi and bacteria [1]. Wood-rotting fungi are the most important producers of laccases, and white-rot basidiomycetes in particular are the only organisms capable of completely degrading lignin, thanks to the coordinated action of a battery of oxidoreductases (ligninolytic peroxidases and laccases), auxiliary enzymes (H_2_O_2_-producing enzymes, cellobiose dehydrogenase, *etc.*) and oxidized species of low-molecular weight compounds (e.g., Mn^3+^, Fe^3+^, ROS, and phenoxyl radicals). Although white-rot basidiomycetes produce the laccases with the highest redox potential in nature, with up to +0.8 V (*vs.* +0.4 to +0.7 V of laccases from bacteria, plants and other fungi), this is still insufficient to oxidize certain components of lignin or other substrates of higher redox potential. However, laccases’ oxidative capabilities can be extended thanks to the presence of redox mediator compounds that, once oxidized by the enzyme, can in turn oxidize a recalcitrant substrate on which laccases cannot act on their own. In addition, mediators may act as electron shuttles, diffusing far away from the laccase active site and oxidizing complex compounds that cannot fit into the substrate binding pocket [2].

Due to their broad substrate range and low requirements, the use of laccases and laccase-mediator systems (LMS) is of great interest for different processes using lignocellulose as renewable raw material, including pretreatment and detoxification of straw materials for the production of second generation bioethanol, biobleaching of fibers in paper and textile industries, deinking of recycled paper or functionalization of lignocellulosic polymers [3,4,5,6,7,8]. Laccases are also endowed with a great potential in organic chemistry for the synthesis of different types of polymers and dyes, naphthoquinones, benzofurans, lignans and other compounds with antioxidant, antimicrobial or antitumoral activities [9,10,11,12,13,14].

Although many LMS applications have been described, these typically rely on synthetic mediators such as ABTS (2,2′-azino-di-[3-ethylbenzthiazoline sulfonate), HBT (1-hydroxybenzotriazole) or violuric acid, which, apart from being expensive, may generate toxic species, hampering their utilization at an industrial scale. For this reason, the search for natural laccase mediators has become increasingly appealing. Indeed, we have described the ability of certain phenolic compounds derived from lignin to act as redox mediators in several works [15,16,17], which is of special interest since these could be easily obtained from lignocellulosic residues or as a byproduct from biomass transformation processes. Some of the most recurrently studied are syringaldehyde, acetosyringone, methyl syringate, vanillin, acetovanillone and *p*-hydroxycinnamic acids (*p*-coumaric, ferulic and sinapic acids) [5,18,19,20,21].

Another bottleneck limiting the industrial use of laccases is the need of high amounts of enzyme, which also needs to be active and stable under the harsh operational conditions. This has encouraged numerous protein engineering efforts in order to heterologously express laccases in different hosts and to obtain tailor-made biocatalysts presenting the desired properties [22]. In previous works, we have described the engineering of two high-redox potential laccase variants from basidiomycete PM1 (PM1-L) and *Pycnoporus cinnabarinus* (PcL), by means of directed molecular evolution in *Saccharomyces cerevisiae* [23,24]. The new laccase variants were actively expressed and secreted by yeast, presented enhanced catalytic efficiencies and retained the stability of the wild-type enzymes. Later on, the two evolved laccase variants were recombined by random DNA shuffling, and selected chimeras presented higher stability, shifted pH activity profiles and better substrate affinities respecting both parents [25]. One of the aforementioned chimeric laccases, variant 3A4, was chosen for its higher affinity towards 2,6-dimethoxyphenol as departure point for further engineering of its activity over lignin derived phenols. In this occasion, saturation mutagenesis was performed over six residues delimiting the substrate binding pocket, and mutant libraries were screened with sinapic acid as model substrate (unpublished data). In the present work, we investigate the oxidation of 28 phenols by selected laccase mutants in order to compare their oxidation according to their different chemical structure and to evaluate how point mutations at the substrate binding pocket might affect laccase activity. The compounds included in this multi-screening assay were chosen for their prevalence in lignin, their ability to act as redox mediators, their use as precursors for added-value products and/or their biological activity as antioxidants, antimicrobials, antitumorals, inmunomodulators, *etc.* [5,7,15,16,17,19,20,21,26,27,28].

## 2. Results and Discussion

**Figure 1:**
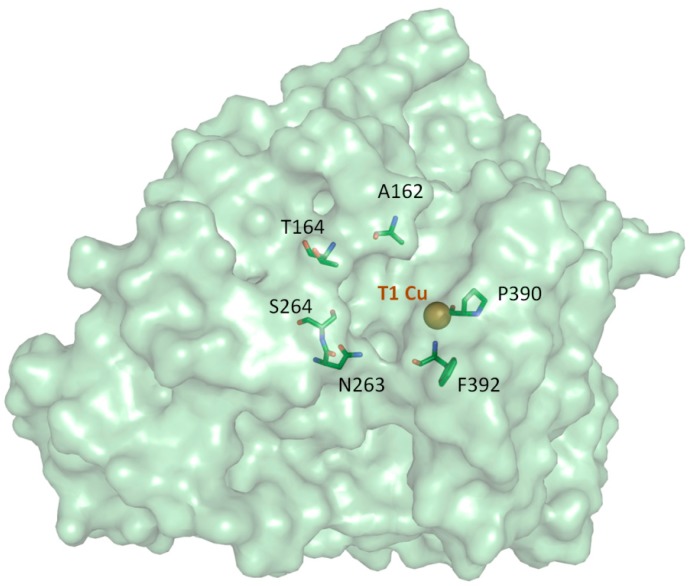
Structure model for 3A4 laccase generated with Swiss-Model server [29], based on the crystal structure of laccase from *Trametes trogii* (PDB 2HRG), with 94% sequence identity. Target residues for mutagenesis are shown as sticks. Catalytic T1 copper is shown as a sphere.

Laccase mutants studied in this work come from the saturation mutagenesis of six residues of the substrate binding pocket of a high-redox potential laccase (3A4) previously engineered in the lab by recombination of PcL and PM1-L [25]. In particular, the residues selected for randomization included Ala162, Thr164, Asn263, Ser264, Pro390 and Phe392 (Figure 1 and Figure S1). These positions were chosen since they were the most variable among the residues delimiting the enzyme pocket in the multiple sequence alignment of laccases from polyporal fungi group to which *P. cinnabarinus* and basidiomycete PM1 belong to (data not shown). 

Three mutant libraries were constructed, each for the combinatorial mutagenesis of two positions: library A, for residues 162 and 164; library B, for residues 263 and 264; and library C, for residues 390 and 392. At least 3066 clones from each library were screened, in order to explore all possible codon-pair combinations with 95% library coverage. Mutant libraries were submitted to a high-throughput screening (HTS) assay based on the activity towards sinapic acid (**14** in Table 1), a lignin related phenol whose oxidation renders a pinkish colored product [24,30]. Clones presenting the highest increases in activity for sinapic acid respecting parent laccase were submitted to a re-screening prior to plasmid extraction, amplification and re-transformation in yeast cells. Thus, 23 laccase mutants in total (seven from library A, four from library B and 12 from library C) and 3A4 parent type were produced in flask cultures in order to obtain sufficient amount of enzyme to evaluate activity towards other phenolic substrates.

**Table 1:** Substrates used for the laccase activity screening of selected mutants. Wavelengths and time-points chosen for each substrate for the calculation of relative activities are indicated.

Code	Name	Structure	Wavelength (nm)	Time (h)
**Phenols**				
**1**	Phenol	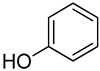	420	24
**2**	Catechol	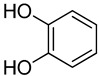	400	2
**3**	Guaiacol	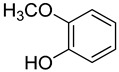	470	2
**4**	Pyrogallol	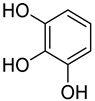	450	2
**5**	2,6-Dimethoxyphenol	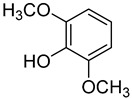	470	0.25 ^a^
***p*-Hydroxybenzoic Acids**				
**6**	4-Hydroxybenzoic acid	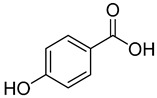	260	24
**7**	3,4-Dihydroxybenzoic acid	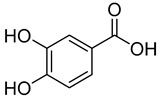	410	2
**8**	Vanillic acid	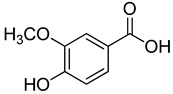	350	4
**9**	Gallic acid	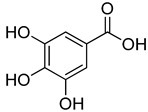	450	2
**10**	Syringic acid	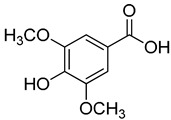	360	2
***p*-Hydroxycinnamic Acids**				
**11**	*p*-Coumaric acid	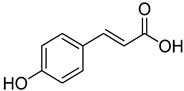	260	24
**12**	Caffeic acid	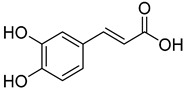	390	2
**13**	Ferulic acid	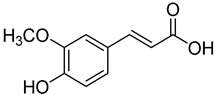	430	2
**14**	Sinapic acid	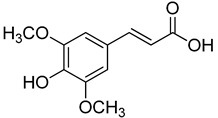	520	2
***p*-Hydroxyacetophenones**				
**15**	4-hydroxyacetophenone	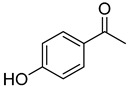	260	24
**16**	Acetovanillone	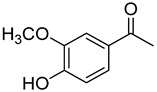	300	24
**17**	Acetosyringone	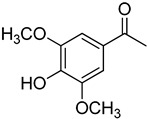	520	8
***p*-Hydroxybenzaldehydes**				
**18**	4-hydroxybenzaldehyde	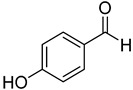	260	24
**19**	Vanillin	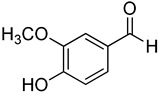	410	24
**20**	Syringaldehyde	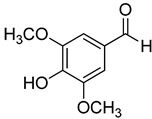	370	4
**Methyl *p*-Hydroxybenzoates**				
**21**	Methyl vanillate	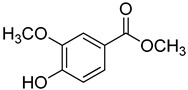	370	24
**22**	Methyl syringate	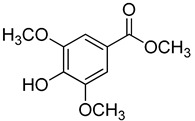	390	1
**Methyl *p*-Hydroxycinnamates**				
**23**	Methyl *p*-coumarate	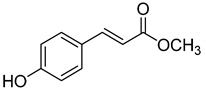	400	24
**24**	Methyl ferulate	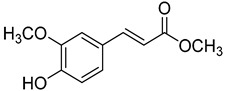	370	1
**25**	Methyl sinapate	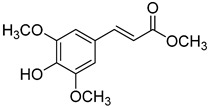	410	4
**Others**				
**26**	Eugenol	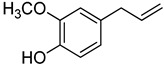	370	1
**27**	Resveratrol	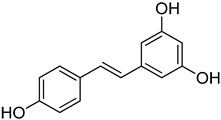	400	1
**28**	Quercetin	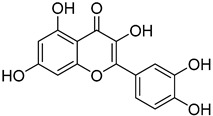	290	4

^a^ Determined in kinetic mode.

### 2.1. Production of Laccase Mutants

**Figure 2:**
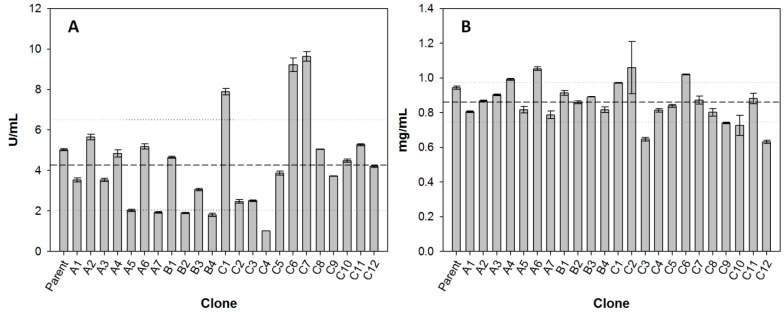
(**A**) Activity measured with ABTS (U/mL) and (**B**) protein concentration (mg/mL) in the concentrated supernatants of the different laccase mutants produced in flask cultures. Error bars represent standard deviation for duplicate measures for each mutant. Dashed lines indicate mean values and dotted lines indicate standard deviation between mutants.

Yeast cells expressing individual laccase mutants were grown in flasks, in expression media for four days, after which cultivations were stopped and laccase activity in the culture media was measured using ABTS as substrate. Surprisingly, several mutants presented remarkable lower activities than the parent type. Protein concentration in the supernatant, however, was more similar between clones (variance coefficient of 14% *vs.* 48% for activity) (Figure 2). 

Due to the mutagenesis strategy used for library construction, the mutations introduced were not expected to significantly affect laccase secretion levels by yeast (as it was lately confirmed, Figure 2b). Hence, the low activities observed in several mutants could be due to (i) a specific enhancement of oxidation of sinapic acid but not of other substrates; or (ii) lower stability of some mutants, which lost activity after longer cultivation times (HTS with sinapic acid was performed two days after inducing laccase expression in microcultures while laccase was induced for four days in flasks).

Concerning the first point, one of the key principles of directed evolution is that “you get what you screen for” [31]. For the engineering of PM1-L and PcL and their chimeric variants, multi-screening assays based on the oxidation of phenolic (2,6-dimethoxyphenol, sinapic acid) and non-phenolic (ABTS) substrates were used in order to preserve the versatility of the parent laccases’ activity [23,24,25]. In this study, however, our goal was to specifically enhance laccase activity towards lignin-derived phenolic substrates. In addition, the high number of clones screened (over 3000 per mutant library) limited the use of high-throughput multi-screening assays for the simultaneous comparison of the three mutant libraries. This way, some selected mutations that are beneficial for activity on sinapic acid may be detrimental for activity on ABTS or other substrates with dissimilar chemical structure. As for the second point, it is well known that in directed evolution approaches there is generally a trade-off between enhanced activity and stability. For this reason, it is recommended to introduce stability assays in order to discard destabilizing mutations. For example, during the directed evolution of PM1-L, a single mutation (F454S) increased parent-type activity fivefold, but it also caused a reduction of T_50_ (10 min) of 5 °C. For this reason, it was removed from the final evolved variant [23,32]. In summary, the differences in activity observed when the selected laccase mutants were produced in flask cultures can be justified by one or both of the explanations accounted for above. In order to compare the activity of the different mutants, we set protein concentration (which was less variable among clones) as the parameter for normalizing the results of the screening of laccase activity with the different phenols.

### 2.2. Multi-Screening with Phenolic Compounds

We selected a battery of 28 compounds as substrates to study their oxidation by chosen laccase mutants produced in flask, following a systematic approach in order to evaluate the effect of the different chemical structures on their feasible oxidation by laccase (Table 1). These comprise phenols with and without substituents in *para*, including carboxyl (free or as methyl esters), ketone, aldehyde and propenoic groups. Besides these substitutions, they can hold different substituents in *ortho* respecting the hydroxyl: one or two hydroxyl groups, one or two methoxyl groups (guaiacyl or G-type and syringyl or S-type, respectively), or none (*p*-hydroxyphenyl or H-type). In addition, three phenolic compounds of renowned interest (eugenol, resveratrol and quercetin) were also evaluated. All these substrates are of plant origin and can be obtained as plant extracts or released during degradation of the lignin polymer either by physicochemical or enzymatic treatment of lignocellulose.

Changes in the absorption spectra of the different phenols in the presence of parent 3A4 laccase along 24 h are shown in Figure S2. In accordance with previous studies, we observed that, in general, substitutions in *ortho* favor phenol oxidation by laccase, being the *o*-di-substituted phenols more easily oxidized than the mono- and non-substituted phenols (the latter were the most recalcitrant). In fact, 4-hydroxybenzoic acid (**6**) was not oxidized at all in the assayed conditions. This is due to the fact that the hydroxyl and methoxyl substituents in *ortho* act as electron donating groups in the aromatic ring (making the π system more nucleophilic) and generally lower the substrate’s redox potential, thus allowing an easier oxidation by laccase. Furthermore, the parent laccase seemed to oxidize phenols with *o-*methoxyl substituents better than with hydroxyl substituents, even though hydroxyls are more activating substituents of the aromatic ring. The same has been described for laccases from *Trametes villosa* and *Lentinula edodes* [33,34], which present higher catalytic efficiency (*k*_cat_/*K*_M_) for guaiacol (**3**) than for catechol (**2**). Similarly, laccase from *Gaeumannomyces graminis* var. *tritici* presented higher affinity (lower *K*_M_) for guaiacol over catechol and for 2,6-dimethoxyphenol (**5**) over pyrogallol (**4**) [35]. This highlights the fact that, apart from a redox potential threshold that must be overcome by laccase, other factors must account for the enzyme’s preference to oxidize certain compounds over others of lower redox potential, mainly a more favorable binding of the substrate. In this sense, the importance of the binding event in the overall efficiency of the enzymatic reaction is influenced not only by steric features that limit the entrance of the substrate to the enzymatic pocket [36], but also by an optimal docking that assures an efficient electron transfer to the T1 Cu site [37].

On the other hand, respecting the effect of the groups/side chains in *para* position, a general tendency (essentially for G- and S-type compounds) was found based on how rapidly the changes in the UV-Vis spectra were observed. The reactivity decreased in the following order: phenols without *p-*substituent > methyl *p*-hydroxycinnamates > *p*-hydroxycinnamic acids > *p*-hydroxybenzoic acids > methyl *p*-hydroxybenzoates > *p*-hydroxyketones > *p*-hydroxyaldehydes. This correlates well with the fact that all the *p*-substituents tested are moderately deactivating, except for the propenoic chain of cinnamic acids and cinnamic esthers due to the presence of the double bond. In fact, during fungal degradation of the lignin polymer, the oxidation of the side chains of the phenylpropane lignin units (Cα-Cβ cleavage) takes place in the initial stages, giving rise to vanillic and syringic acid as main degradation products from G- and S-type units, respectively [38].

### 2.3. Comparison of Laccase Mutants

In order to compare the activity of the different laccase mutants, an appropriate wavelength was chosen for each substrate. When changes in the absorption spectra were observed in more than one peak, increase in the visible wavelength range was selected, since these could be useful for developing future colorimetric high-throughput screening (HTS) assays. To detect differences between clones, time points were selected so that the change in absorbance (Δ*Abs*) observed for the parent type at the given wavelength was around 50% of the maximum Δ*Abs*. Relative activity for each clone respecting parent type was calculated according to the formula:

(1) $$\left(\left(\Delta {\mathrm{Abs}}_{\text{X}}\text{ - }\Delta {\mathrm{Abs}}_{\mathrm{blank}}\right)/{\left[\mathrm{protein}\right]}_{\text{X}}\right)/\left(\left(\Delta {\mathrm{Abs}}_{\mathrm{parent}}\text{ - }\Delta {\mathrm{Abs}}_{\mathrm{blank}}\right)/{\left[\mathrm{protein}\right]}_{\mathrm{parent}}\right)$$

**Figure 3:**
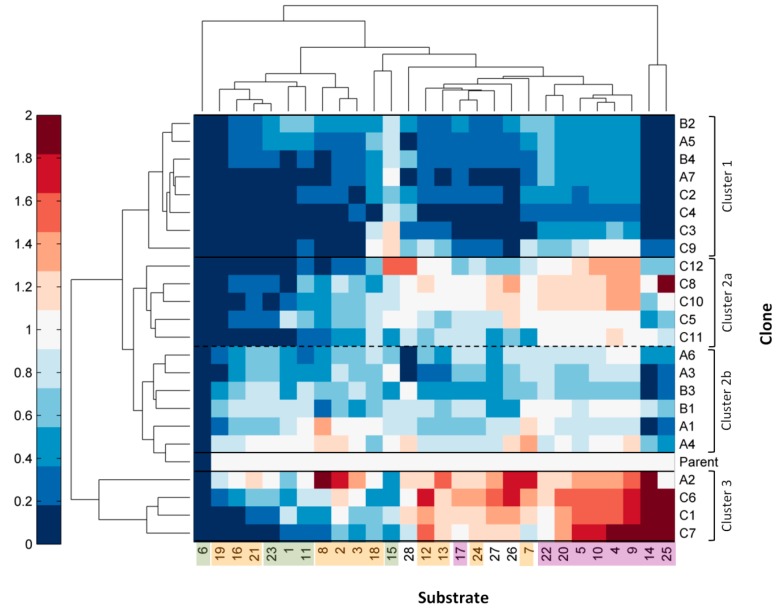
Heat map with hierarchical clustering based on the relative activity values (respecting parent type laccase) of the library of laccase mutants (rows) towards the different phenols under study (columns). Substrate labels are highlighted in purple for *o*-di-substituted phenols, orange for *o*-monosubstituted phenols, green for H-type phenols, and non-highlighted for others. The three main clusters grouping the laccase mutants are indicated.

Results for relative activities were represented in a heat map and clustered according to substrate (columns) and laccase mutant (rows) (Figure 3). *o*-Di-substituted phenols, for which the highest increases in activity were found, were grouped together at the right side of the map. In fact, sinapic acid and methyl sinapate (**14** and **25**, respectively) formed a distinct cluster, separate from the rest of substrates tested. The utmost enhancement of laccase activity on **14** and **25** is expected because laccase mutants were selected based on their activity towards sinapic acid, and methyl sinapate is the most structurally similar compound used in the screening. *o*-Monosubstituted phenols, on the other hand, were more evenly represented in the different clusters. It is worth noting that vanillin related compounds (**16**, **19** and **21**) clustered at the far left side of the map, with laccase mutants presenting even worse activity for these substrates than towards the theoretically more difficult to oxidize H-type phenols (except for **6**). Interestingly, greater increases in activity were found for substrates with hydroxyl substitutions in *ortho* than for those with methoxyl substitutions (except for **7** and **8**), even though parent laccase was more efficient oxidizing the latter. The mutations introduced in the enzyme binding pocket are not expected to significantly affect the laccases’ redox potential, since none of the target residues coordinate the T1 Cu (the closest are Pro390 and Phe392, located in the same loop as His395 that coordinates T1 Cu). For this reason, the observed differences between mutants are most probably caused by different affinities. In fact, it is possible that not all substrates were used in saturating concentrations in the screening assay and, consequently, oxidation rates would be affected by the mutants’ affinity towards the different compounds.

Respecting laccase mutants, roughly three main clusters were formed. Cluster 1 was formed by clones that performed worse than parent type laccase towards most substrates (upper part of the map). Except for the case of clone C9, mutants grouping in this cluster are those that presented lower activity towards ABTS in the culture supernatant (Figure 2a), meaning that these laccase variants were generally less active than the parent type. The second cluster included clones that retained similar activity values than parent type, and it could be subdivided into a group which presented a general enhanced oxidation of phenols with two substitutions in *ortho* (Cluster 2a, mutants from library C) and another group that did not (Cluster 2b, mutants from libraries A and B). Finally, Cluster 3 was formed by four clones presenting the highest increases in activity towards several compounds (lower part of the map). These included three mutants from library C and one from library A. They showed enhanced oxidation over all *o*-di-substituted phenols tested and several *o*-monosubstituted phenols (including eugenol) and, interestingly, also increased activity on resveratrol.

Analysis of DNA sequences revealed that all clones from library C mutated Phe392 for other residues. In particular, clones belonging to Cluster 3, namely C7, C1 and C6, mutated Phe392 for Asn, Thr and Gln, respectively, but maintained Pro390 from parent type. It is worth noting that the best mutants from cluster 2a, C8 (F392S) and C10 (F392V), which also presented better activity towards *o*-di-substituted phenols but to a lesser extent, also maintained parent Pro390. The mutations found in these clones revealed that while position 392 is quite flexible, accommodating variable residues, position 390 is more restrictive. As for A2 mutant, substitutions A162K and T164I were found. Remarkably, clone A2 was significantly better at oxidizing *o-*monosubstituted phenols (e.g., **2**, **3**, **8**, **7** and **13**) than di-substituted phenols, in comparison to clones from library C. The same occurred for clones A1 (A162K T164V) and A4 (A162R). This suggests that the substitution of Ala162 for basic residues Lys or Arg could be beneficial for the oxidation of this type of compounds. Interestingly, several studies on laccase structure–function relationships hypothesize that a nonpolar residue in position 162 is important to establish correct hydrophobic interactions with the phenolic substrate [39,40]. However, our results point in the opposite direction, at least for the oxidation of phenolic substrates with one or two hydroxyl or methoxyl substituents in *ortho.* This highlights the often-difficult prediction of amino acid substitutions to enhance enzyme activity by rational design, and by contrast, the potential of directed evolution and random approaches to reveal unexpected mutations that might provide the solution to a problem.

## 3. Experimental Section 

### 3.1. Construction and Screening of Mutant Libraries

General considerations for DNA manipulation and library screening are described elsewhere [23,24,25]. Mutant libraries were obtained by in Vivo Overlap Extension (IVOE) [41]. Amino acids in positions 162, 164, 263, 264, 390 and 392 were randomized using mutagenic primers with NNK degeneracy. Each pair of mutagenic primers simultaneously mutated two nearby residues, so that three individual libraries were obtained: library A (positions 162 and 164), library B (positions 263 and 264) and library C (positions 390 and 392). The 5′-end gene fragments were amplified with RMLN and reverse mutagenic primers, and the 3′-end fragments with RMLC and forward mutagenic primers, using pJRα3A4 as template [30]. Purified PCR products were co-transformed in yeast together with the linearized expression vector (pJRoc30) using Yeast Transformation Kit (Sigma, St. Louis, MO, USA). Individual colonies were picked and cultured in 96-well format in minimal media for two days and in expression media for another two days [23,24,25]. Then, plates were centrifuged and 20 μL aliquots of supernatant were transferred to new plates for screening, which was performed by adding 180 μL of 250 μM sinapic acid in 100 mM acetate buffer, pH 5.0. Transformation of sinapic acid by laccase was followed in end-time mode in the plate reader, measuring the increase in absorption at 512 nm [24,30]. Mutants presenting higher activity were submitted to a re-screening, in which activity of each mutant was measured in quadruplicate. Plasmids from clones selected in the re-screening were extracted and transformed in *E. coli* strain DH5α in order to obtain sufficient copies for DNA sequencing and for re-transformation in yeast cells.

### 3.2. Production of Laccase Mutants

Re-transformed yeast cells expressing laccase mutants selected in the re-screening were produced in duplicate in 100 mL flasks containing 30 mL of expression media [23,24,25], growing the cells at 30 °C, 220 rpm, for 4 days. Afterwards, cultures were centrifuged at 5000 RCF (Relative Centrifugal Force) for 20 min and supernatants were sequentially filtered through 0.8 and 0.45 μm pore-size membranes. Filtered supernatants were then concentrated tenfold in Amicon Ultra 10000 MWCO centrifugal units (Millipore, Darmstadt, Germany). Laccase activity in the concentrated extracts was measured with 3 mM ABTS (ε_418_ = 36000 M^−1^·cm^−1^) in 100 mM acetate buffer, pH 5.0, and protein concentration was determined with the Bio-Rad Protein Assay, using the microassay procedure for microtiter plates.

### 3.3. Multi-Screening with Phenolic Compounds

All the assayed substrates were purchased from Sigma-Aldrich, except for methyl syringate, which was purchased from Alpha Aesar (Karlsruhe, Germany), and methyl coumarate, methyl ferulate and methyl sinapate, from Apin Chemicals (Abingdon, UK). Stock solutions for the different phenols were routinely prepared at 10 mM in 20% ethanol, except for phenol, catechol, pyrogallol, guaiacol and 2,6-dimethoxyphenol, which were dissolved in water; and resveratrol and quercetin, which were dissolved in absolute ethanol. Reactions were carried out in duplicate for each mutant in UV-Star 96-well plates (Biogen, Cambridge, MA, USA), by adding 10 μL of laccase crude extracts, 180 μL of 100 mM acetate buffer, pH 5.0, and 20 μL of stock substrate solution in each well. Control reaction without enzyme was included in order to subtract non-enzymatic substrate oxidation. UV-Vis absorption spectra (250–700 nm, with steps of 10 nm) were recorded in a SpectraMax Plus 384 plate reader (Molecular Devices, Sunnyvale, CA, USA) at reaction times 0, 30 min, 1 h, 2 h, 4 h, 6 h, 8 h, and 24 h. In the case of 2,6-dimethoxyphenol, reaction was too fast, so it was measured in kinetic mode at 469 nm (ε_469_ = 27500 M^−1^·cm^−1^). For each substrate, absorption peaks and appropriate time points were selected. Average ΔAbs per mg of protein was calculated for each mutant and normalized against parent type. Results were represented in a heat map and hierarchical clustering analysis was performed using the clustergram algorithm with Matlab software (MathWorks, Natick, MA, USA).

## 4. Conclusions 

In this work, we have evaluated the oxidation of a battery of phenols with different chemical structures by a library of laccase mutants obtained by saturation mutagenesis of six residues of the substrate binding pocket. This has given us some insight into the structural factors, both from the substrate and the enzyme, that modulate laccase activity. Even though the substrates tested are structurally related to sinapic acid, high variability in laccase activity was found only by changing one or two residues of the enzyme pocket. This indicates that besides the difference in redox potential between laccase and substrate, which delimits the electron transfer, the efficient binding of the substrate to the enzymatic pocket determines the oxidative capabilities of laccases. These results also highlight once more the importance of the screening assay for directed evolution, which will define the fate of the protein subject of the study. The use of either a single or a multi-screening assay will determine whether we obtain a specialist or a generalist biocatalyst, respectively. 

## Supplementary Materials

Supplementary materials can be accessed at: http://www.mdpi.com/1420-3049/20/09/15929/s1.
