# Supplementary material for: Exploring the Oxidation of Lignin-Derived Phenols by a Library of Laccase Mutants
Source: Molecules. 2015 Sep 2;20(9):15929–43. doi: 10.3390/molecules200915929 (PMC6332420; doi:10.3390/molecules200915929)
Supplement: Supplementary file 1 [file molecules-20-15929-s001.pdf]

Supplementary Materials

SIGPVADLTISNGAVSPDGFSRQAILVNDVFPSPLITGNKGDRFQLNVIDNMTNHTMLKS 60
TSIHWHGFFQHGTNWADGPAFVNQCPISTGHAFLYDFQVPDQAGTFWYHSHLSTQYCDGL 120
RGPIVVYDPQDPHKSLYDVDDDSTVITLADWYHLAAKVGPAAPTADATLINGLGRSINTL 180
NADLAVITVTKGKRYRFRLVSLSCDPNYTFSIDGHSLTVIEADGVNLKPQTVDSIQIFPA 240
QRYSFVLNADQDVDNYWIRALPNSGTRNFDGGVNSAILRYEGAAPVEPTTTQTPSTQPLV 300
ESALTTLEGTAAPGNPTPGGVDLALNMAFGFAGGRFTINGASFTPPTVPVLLQILSGAQS 360
AQDLLPSGSVYSLPANADIEISLPATSAAPGFPHPFHLHGHTFAVVRSAGSSTYNYANPV 420
YRDVVNTGSPGDNVTIRFRTDNPGPWFLHCHIDFHLDAGFAVVMAEDTPDTKAANPVPQA 480
WSDLCPIYDALDPSDL 496

Figure S1. Amino acid sequence of chimeric 3A4 laccase used as parent type in this work. Sequence inherited from PcL evolved variant during chimeragenesis is underlined (residues 457–496), while the rest corresponds to PM1-L evolved variant. Copper coordinating residues are highlighted in cyan. Mutated residues located at the substrate binding pocket are shown in magenta.

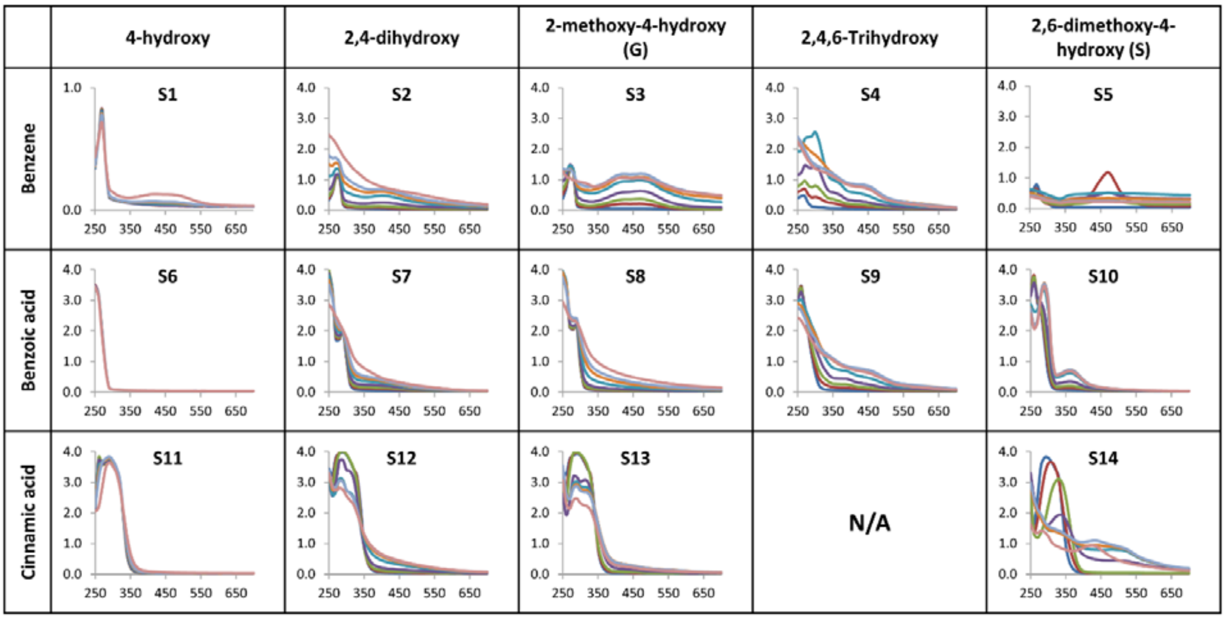

Figure S2. *Cont.*

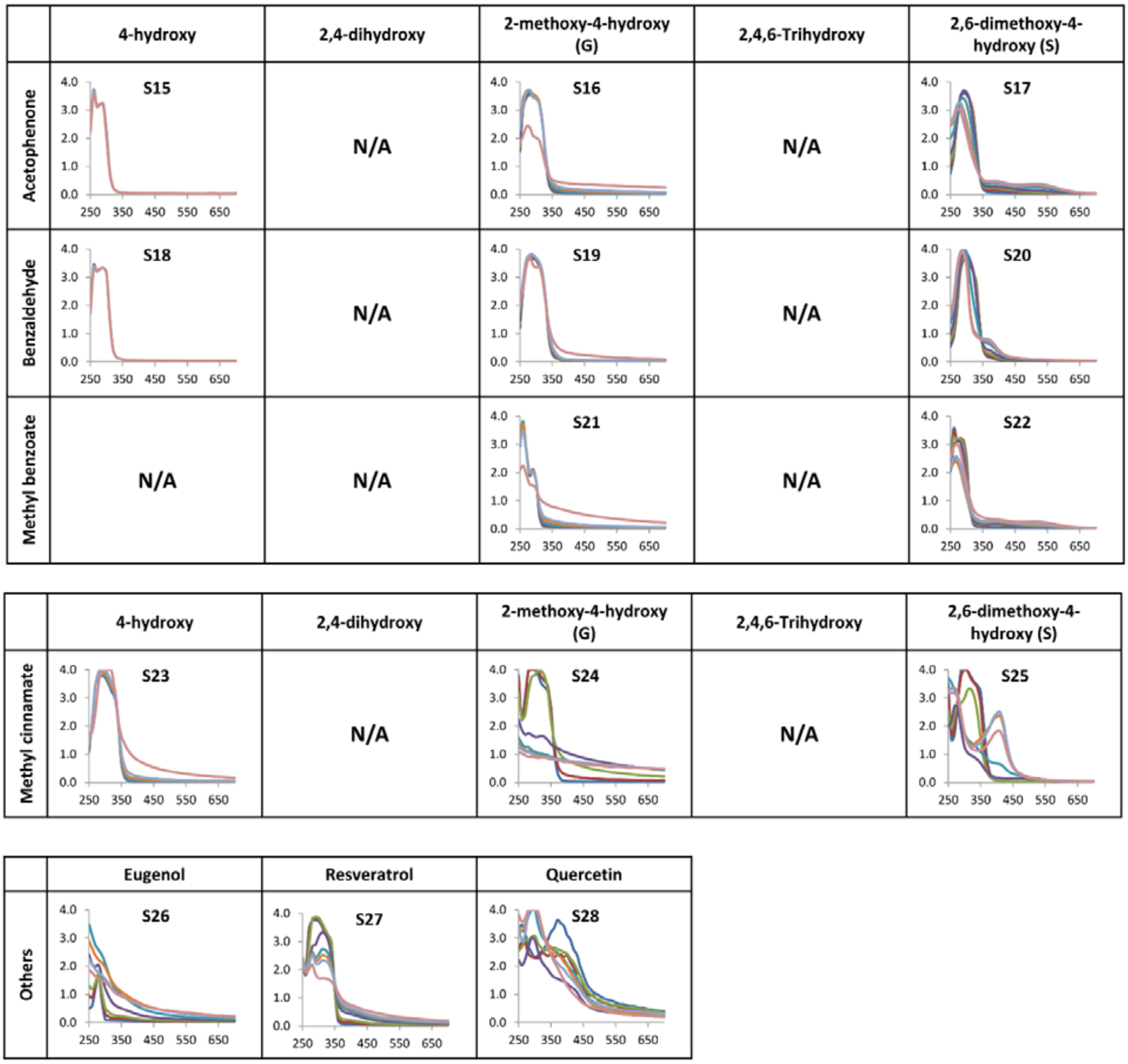

Figure S2. Changes in UV-Vis absorption spectra of the different phenols upon oxidation by laccase. Spectra shown correspond to initial time (**blue**), 30 min (**red**), 1 h (**green**), 2 h (**purple**), 4 h (**cyan**), 6 h (**orange**), 8 h (**light blue**) and 24 h (**pink**).
